# Leveraging AHP and transfer learning in machine learning for improved prediction of infectious disease outbreaks

**DOI:** 10.1038/s41598-024-81367-1

**Published:** 2024-12-31

**Authors:** Reham Abdallah, Sayed Abdelgaber, Hanan Ali Sayed

**Affiliations:** 1 Information Systems Department, Faculty of Computers and Artificial Intelligence, Helwan University, Cairo, Egypt; 2 Public Health and community medicine Department, Theodor Bilharz Research Institute, Helwan University, Cairo, Egypt

**Keywords:** Infectious diseases, AHP, Transfer learning, Risk factors, Machine learning, Biotechnology, Diseases, Risk factors

## Abstract

Infectious diseases significantly impact both public health and economic stability, underscoring the critical need for precise outbreak predictions to effictively mitigate their impact. This study applies advanced machine learning techniques to forecast outbreaks of Dengue, Chikungunya, and Zika, utilizing a comprehensive dataset comprising climate and socioeconomic data. Spanning the years 2007 to 2017, the dataset includes 1716 instances characterized by 27 distinct features. The researchers adopt the Analytic Hierarchy Process (AHP) for feature selection and integrated transfer learning to boost the accuracy of the study’s predictions. The researchers’ approach involves the deployment of several machine learning algorithms, including Random Forest, XGBoost, Gradient Boosting, and an ensemble of these methods. The result reveals that the ensemble model is particularly effective, achieving the highest accuracy rate of 96.80% and an AUC of 0.9197 for predicting Zika outbreaks. Furthermore, it exhibts consistent performance across various metrics. Notably, in the context of Chikungunya, this model achieves an optimal balance between precision and recall, with an accuracy of 93.31%, a precision of 57%, and a recall of 63%, highlighting its reliability for effective outbreak prediction.

## Introduction

Infectious diseases continue to pose a significant threat challenge to global health, causing millions of deaths annually and disrupting societies^1^. An outbreak is defined as an unexpected surge in disease cases that exceed the expected levels within a particular geographic area or demographic group^2^. Early and precise prediction of such outbreaks is essential for public health authorities to deploy effective control and prevention strategies, thereby mitigating impacts on public health and resources^3^. Despite this, the complicated dynamics of infectious diseases, combined with limitations in available data, pose significant challenges to traditional prediction methods. Although explicit prevention of outbreaks may not always be feasible, it is entirely possible to prepare adequately for them. The development and deployment of intelligent predictive models are rapidly advancing, significantly enhancing both individual healthcare and global disease management. Modern machine learning models facilitate the monitoring and prediction/forecasting disease case numbers. Increasingly, these models support medical professionals in diagnosis, treatment and aid public health strategies designed to prevent disease spread globally^4^. A key step in mobilizing healthcare responses to impending outbreaks is visualizing and evaluating outbreak predictions^5^. Despite significant advancements in healthcare, infectious diseases continue to be a formidable threat to global health, with epidemics capable of causing extensive morbidity and mortality. Machine learning (ML) has emerged as a powerful tool, capable of analyzing various and diverse datasets to identify complex relationships among the various factors that influence disease dynamics. Nevertheless, obstacles such as data scarcity, task complixity, and the robustness of evaluation methodologies persist. ML algorithms exceed in identifying patterns and trends within complex data sets that may fail human detection. By continuously monitoring risk factors like seasonal variations and travel patterns, ML enables the timely prediction of outbreaks, allowing for early interventions that can effectively allocate resources and prevent minor outbreaks from intensifying into major epidemics. The analytic hierarchy process (AHP), a multi-criteria decision making (MCDM) approach developed by Thomas L. Saaty, organizes factors into a hierarchical structure, proving invaluable for decision-makers navigating complex scenarios. This approach assists in defining priorities and assigning weights to various factors by establishing relevant criteria and constructing a pairwise comparison matrix to ensure decision consistency in making a decisionor in decision making^6^. The AHP utilizes a fundamental scale ranging from 1 to 9 to assess the relative importance of two items, facilitating the calculation of each factor’s weight in the decision-making process^7^. The accuracy and potential biases within the decision-making process are verified through the consistency index (CI) and consistency ratio (CR); a CI to random consistency index (RI) ratio exceeding 0.1 indicates inconsistency^8^. Understanding the primary risk factors for infectious diseases is cruical for mitigating outbreaks. A comprehensive analysis of socioeconomic, demographic, geographic, climatic, behavioral, and health-related factors reveals their significant impact on disease patterns^2^. Environmental variables such as temperature, humidity, seasonal changes, hygiene practices, population age distribution, and urban economic conditions play a critical role in influencing disease transmission and spread^9^. In real-world scenarios, such as infectious disease outbreak prediction, constructing models from scratch or collecting new training data is often exorbitantly costly or impractical. Thus, minimizing data collection efforts is essenial. Transfer learning, which utilizes knowledge from one domain to solve related issues in another, addresses this by transferring models, weights, or features from one disease context to another^10,11^. This study uses Zika, Chikungunya, and Dengue as case studies to demonstrate the approach, presenting an enhanced model for infectious disease outbreak prediction. By integrating the Analytical Hierarchy Process with Machine Learning, the model systematically identifies and prioritizes risk factors, considers their interconnections, and applies these insights across multiple diseases via transfer learning to enhance prediction accuracy and reliability.

## Literature review

Recent studies that apply the analytic hierarchy process (AHP) in the context of disease or outbreak prediction include the following:

G Harsha et al. (2023) employ both the analytic hierarchy process (AHP) and the fuzzy-AHP (F-AHP) to determine dengue risk zones in Kerala. Their methodology incorporat geo-environmental and demographic variables, including normalized difference vegetation index (NDVI), land surface temperature (LST), topographic wetness index (TWI), land use land cover (LULC), elevation, normalized difference built-up index (NDBI), household density, and population density. The findings indicate that the F-AHP model outperformed the traditional AHP in predictive capability, achieving an AUC of 0.971. Additionally, it is established that the zones classified as extremely high and high risk accounted for approximately 82.87% of the reported dengue cases^12^.

E. Badillo–Rivera et al. (2020) investigat the social and environmental determinants influencing the transmission of the SARS-CoV-2 virus. Utilizing the Analytic Hierarchy Process (AHP) and Geographic Information System (GIS), they identify susceptible regions within Peru. The study determins a consistency ratio (CR) of 0.032, indicating reliable analytical outcomes. Results demonstrat that approximately 68% of Peruvian regions fall into ‘high’ or ‘very high’ risk categories for the spread of the SARS-CoV-2 virus^13^.

A Fariza et al. (2021) introduce a novel approach for assessing diphtheria susceptibility in East Java Province, Indonesia, by integrating the analytic hierarchy process (AHP) with the natural breaks classification system. This methodology classifies diphtheria vulnerability into three distinct tiers: low, medium, and high. Utilizing the combined AHP and natural breaks classification approach, the study demonstrats high accuracy with a value of 0.77^6^.

R Mahato et al. (2020) employ the analytic hierarchy process (AHP) in conjunction with Geographic Information Systems (GIS) to identify potential COVID-19 risk zones across several Indian states. Their findings suggest an elevated risk of increased infection rates particularly in the central regions of India. Key factors contributing to this risk include the number of confirmed cases, the proportion of the population living below the poverty line, and the percentage of urban population^7^.

Sk Ajim Ali et al. (2019) apply the analytic hierarchy process (AHP) alongside geographic information systems (GIS) to analyze mosquito-borne disease distribution, incorporating various environmental factors as decision-making criteria. These factors includ surface temperature, Normalized Difference Vegetation Index (NDVI), land cover, vegetation, land elevation, and slope. The study’s findings underscore a high degree of consistency in decision-making, with the consistency ratio (CR) consistently below 0.1, indicating robust reliability. Among the evaluated factors, water bodies are identified as presenting the highest risk for disease transmission^8^.

SA Ali et al. (2018) integrat environmental data to delineate dengue risk zones in Kolkata, India, using a two-stage approach involving geographic information systems (GIS) and the analytic hierarchy process (AHP). The first stage involves using GIS to analyze environmental factors, followed by the application of AHP in the second stage to assess the correlation between these environmental elements and dengue risk zones. The study concludes that increased building densities, population densities, and high concentrations of people in confined areas significantly contribut to the rise in dengue fever incidence^14^.

An analysis of prior research reveals that existing studies predominantly focus on predicting specific diseases using open-source libraries like scikit-learn for feature selection. However, these libraries are often predefined and may not fully accommodate the unique attributes of diverse datasets, potentially leading to inefficient feature selection and data transformation. This study seeks to overcome these challenges by implementing the analytic hierarchy process (AHP) for more precise feature selection, thus aiming to bridge the identified gaps and enhance the accuracy of disease prediction models. Moreover, the scarcity of labeled data presents a significant obstacle in predictive modeling for disease epidemics. To address this, the researchers’ approach incorporates transfer learning, utilizing pre-trained models from large, related datasets to support the effective utilization of existing data and improve performance on targeted tasks.

## Research methodology

**Fig. 1:**
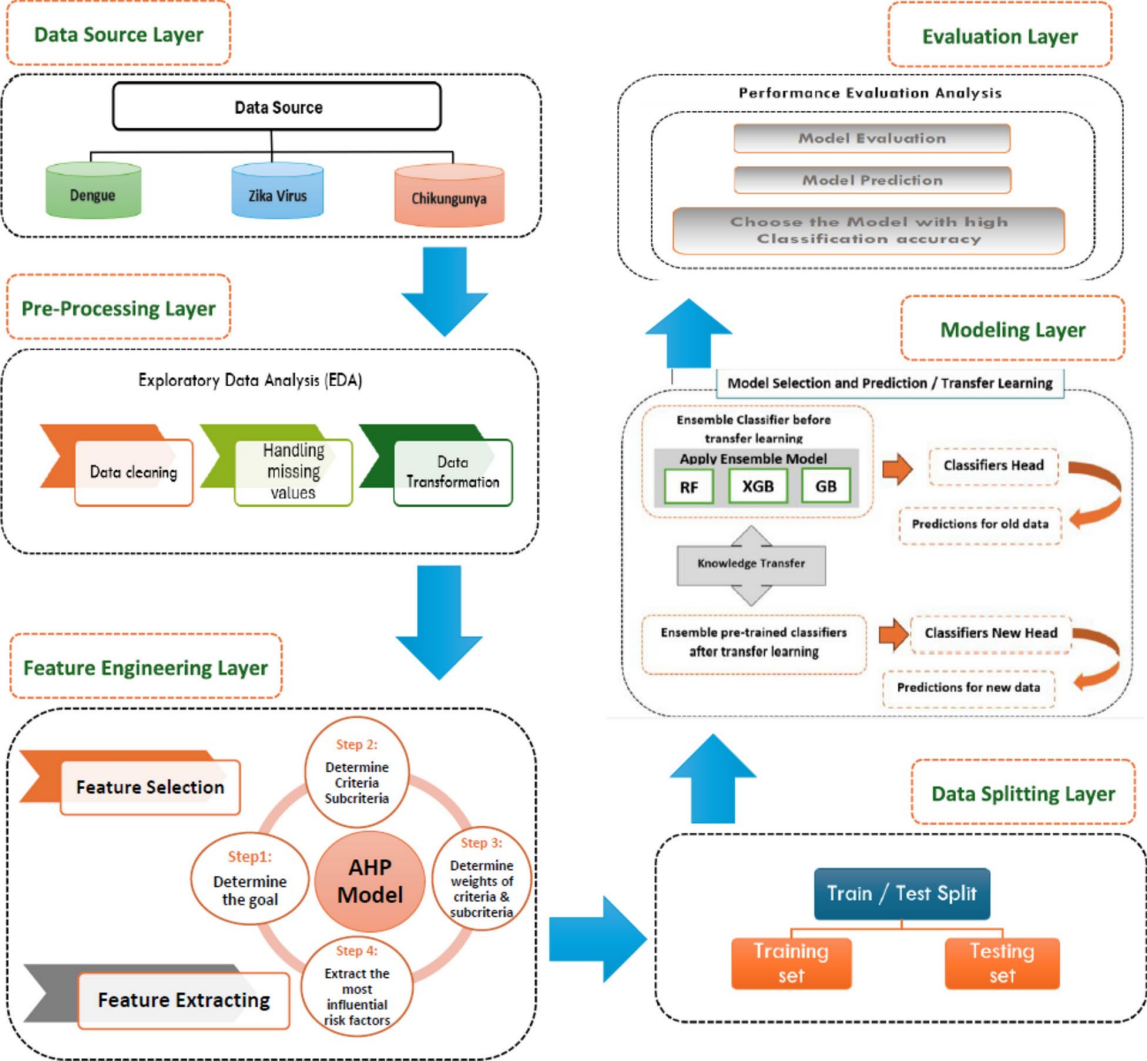
Proposed model for epidemic prediction using AHP and Ensamble machine learning.

Mosquito bites are a major vector for transmitting diseases that can lead to epidemics, particularly in tropical and subtropical regions^15^. Accordingly, this paper introduces a methodological model that integrates the Analytic Hierarchy Process (AHP) for feature prioritization with advanced ensemble machine-learning techniques to anticipate potential outbreaks of epidemic diseases. The researchers specifically focus on Dengue, Zika, and Chikungunya, which are among the fastest-growing viral diseases globally, transmitted by female Aedes mosquitoes^15^. The proposed model consists of six layers: the data source layer, preprocessing layer, feature engineering layer, data splitting layer, modelling layer, and evaluation layer, as illustrated in Figure 1. To identify common risk factors associated with infectious disease outbreaks, this study employes a predefined search technique across major online databases including PubMed/Medline, Scopus, and CINAHL, using search terms such as outbreak*, epidemic*, pandemic*, emerging disease*, and re-emerged disease*.

### Data source layer (data acquisition)

Various datasets are utilized to implement the proposed model. Initially, climate datasets collected from 2007 to 2017 are collected from the NASA Langley Research Center, DANE (National Administrative Department of Statistics of Colombia), and SIVIGILIA (National Public Health Surveillance System, Colombian National Institute of Health).

These datasets encompass both climate and socioeconomic data, detailed as follows:

Climatic data: this dataset provides extensive information on climatic variables for each municipality, including average temperature (tavg), minimum temperature (tmin), maximum temperature (tmax), average humidity (havg), wind speeds (maximum (wsmax), minimum (wsmin), and average (wsavg)).

Socioeconomic data: this includes sociodemographic indicators critical for public health analysis, such as illiteracy, low educational achievement, multidimensional poverty index (mpi), child labor, school absence, informal work, school lag, population, lack of health insurance, and dependency rate.

Upon integration of these data, the final dataset comprises 1716 entries, focusing on the diseases dengue, chikungunya, and zika, each distinguished by 27 unique features.

### Data pre-processing layer

This layer encompasses exploratory data analysis (EDA), which addresses initial errors, missing values, and inconsistencies in the dataset. The EDA process transforms the data into a suitable format for feature engineering and predictive modeling, ensuring that the dataset is cleaned and standardized for subsequent analysis stages.

#### Data cleaning

During this phase, the researches addresse error detection, including the identification of negative case counts and misclassified disease types. Additionally, outliers are removed by calculating the Interquartile Range (IQR) for each variable. Values identified as outliers are those that fall below Q1 − 1.5 IQR or exceed Q3 + 1.5 IQR.

#### Handling missing values

To preserve the integrity of the dataset for analysis, it is essential to address the missing values using appropriate correction or imputation techniques. For instance, the missing values for the average temperature (Tavg) feature are replaced with the mean temperature calculated from the available temperature records.

#### Data transformation

In this phase, Min–Max normalization is employed to scale continuous data, including population metrics, temperature, and precipitation, to a uniform range between 0 and 1. Additionally, one-hot encoding is applied to convert categorical variables, such as “Municipality,” into a numerical format represented by binary indicators.

### Feature engineering layer

Feature Engineering is a critical step in developing predictive models. It involves selecting the most relevant features and constructing new variables to enhance both model accuracy and interpretability. This process encompasses feature selection and feature extraction, both of which are vital for effectively preparing the data for modeling.

### Feature selection

Feature selection involves identifying and selecting most pertinent characteristics for forecasting outbreaks^16^. In this phase, a semi-automated process is employed to optimize feature selection. The Researchers’/the study’s predictive model integrates the systematic consistency of the analytic hierarchy process (AHP) with expert domain knowledge to calculate weights for all diseases collectively. This approach utilizes the combined insights from various diseases to identify the most influential features, ensuring their relevance across different contexts. The steps of the AHP model are as follows^17^:

Step 1: Determine the goal of the AHP model.

Identify the most influential risk factors of infectious disease outbreaks.

Step 2: Determine criteria/sub-criteria.

Identify criteria such as climate factors, population demographics, and socioeconomic elements. These criteria are further decomposed into various components that influence the spread of disease.

Step 3: Expert input and pairwise comparisons:

#### Expert manual input

Expert manual input serves as the foundation for the data used in the AHP model. Domain experts evaluate potential features based on their knowledge and familiarity with the epidemiological factors influencing disease outbreaks. They assign initial weights reflecting the significance of each feature’s impact on disease transmission. Subsequently, these manual input weights are normalized using the following equation:

$$Normalized\,Weight = \frac{Given\,Weight}{{Sum\,of\,all\,Given\,Weight}}$$

#### Conducting pairwise comparisons

In this step, pairwise comparisons are employed to derive standardized, objective weights for each feature based on the manually assigned weights. Utilizing the AHP technique, pairwise comparisons is created among all criteria and sub-criteria. Each pair of factors is evaluated to determine which is more significant, specifically in the context of outbreak prediction^18,19^.

Step 4: calculation of weights and consistency checking: This step ensures that each factor’s relative importance is accurately represented. A consistency check is then performed to validate the consistency of the judgments, ensuring the reliability of the weight assignments.

Step 5: conduct a consistency ratio (CR) evaluation: to verify the reliability and consistency of expert inputs and the pairwise comparison process^18^.

Step 6: Extract the weighted priorities of the selected risk factors.
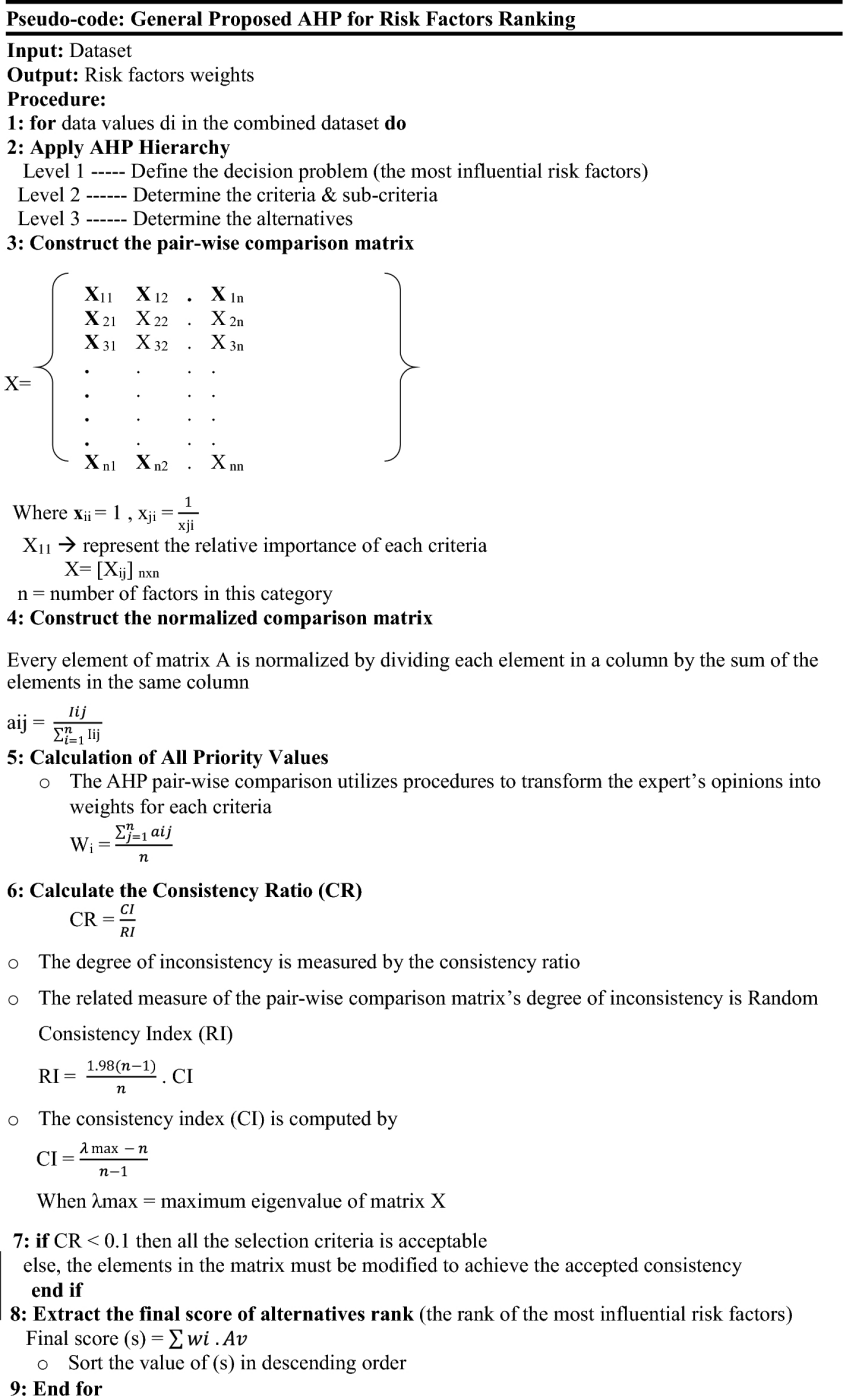


In the final AHP model, de a formula is developed for calculating risk factor weights. This formula includes multiple factors, each multiplied by a weight derived from the correlation confusion matrix. To accurately assess each factor’s significance, the AHP weights are calculated by integrating expert opinions with the correlation coefficient matrix. This approach enhances this model’s ability to rank epidemiological risk factors effectively, thereby improving its capacity to predict potential disease outbreaks. Table 1 below represents the weight of each risk factor, ordered by highest ranking.

**Table 1:** Risk factors ranks.

Weight	Risk factor	Rank
0.667	Barriers to health services	1
0.350	Dependency rate	2
0.333	No health insurance	3
0.307	Barriers to childhood services	4
0.307	Precipitation	4
0.237	Informal work	6
0.218	School absence	7
0.218	Elevation	7
0.159	Child labour	9
0.154	WSavg	10
0.154	School lag	10
0.109	Wsmin	12
0.109	Municipality	12
0.106	Low educational achievement	14
0.076	Wsmax	15
0.076	Critical overcrowding	15
0.070	Illiteracy	17
0.053	Havg	18
0.053	Inappropriate wall exterior	18
0.046	MPI	20
0.037	Tmax	21
0.037	Inappropriate flooring material	21
0.032	Population	23
0.026	Tavg	24
0.026	Inadequate excreta disposal	24
0.019	No access improved water	26
0.019	Tmin	26

**Fig. 2:**
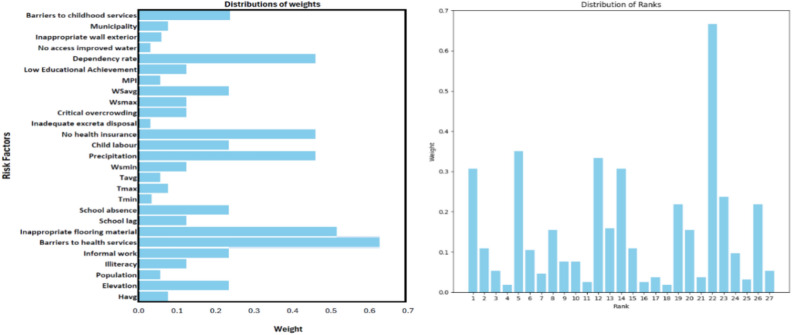
Distributions of AHP weights and ranks for risk factors.

Based on the previous rankings of risk factors shown in Fig. 2, it is concluded that the highest-ranked factors; barriers to health services, dependency rate, and lack of health insurance play a critical role in disease outbreaks. Other notable factors, such as barriers to childhood services, precipitation, and informal employment, reflect important socioeconomic and environmental influences. Conversely, lower-ranked factors like population, average temperatures (Tavg, Tmax, Tmin), and inadequate excreta disposal have comparatively lesser impact on predicting outbreaks. This ranking aids in prioritizing the most influential factors, thereby enhancing efforts in disease prevention and control.

### Feature extraction

Feature extraction involves transforming raw data into a format more suitable for modeling, a crucial step in enhancing the dataset’s predictive power for disease outbreaks. In this study, the dataset initially lacked classification and a defined target variable. To address this issue, the 75th percentile method is used to establish an outbreak threshold, effectively distinguishing between normal conditions and outbreak situations and creating a target variable from disease incidence data. This statistical approach analyzes historical disease occurrence rates to identify a critical value that indicates an unusually high incidence of disease. By setting the threshold at the 75th percentile, it is ensured that only the top 25% of data points, representing unusually high incidences, are classified as outbreaks. To compute the 75th percentile of the dataset, the researchers use the standard percetiled formula:

$$P = \frac{n + 1}{{100}} \times 75$$

where:

P = Percentile value.

n = Total number of observations.

After establishing the threshold, the final outbreak target is calculated, a binary variable indicating the presence or absence of an outbreak. If disease incidence exceeds the defined threshold, the outbreak target is set to 1 (indicating an outbreak); otherwise, it is set to 0 (indicating no outbreak).

### Data splitting layer

In this phase, the dataset is split into 80% for training and 20% for testing to assess the model’s predictive accuracy on unseen data. This approach allowes the researchers to build and validate a model initially trained on Dengue data, which is then evaluated with data from Zika and Chikungunya viruses.

### Modeling layer

After feature selection and data preprocessing, transfer learning is applied to use Dengue data as a base model for forecasting Zika and Chikungunya outbreaks. The model is developed by using Random Forest, XGBoost, and Gradient Boosting algorithms. Additionally, an ensemble technique is employed to combine predictions from these individual models, enhancing overall predictive performance. The algorithm below details the outbreak prediction process using transfer learning. Note that the dataset is imbalanced due to unequal distribution of target classes.
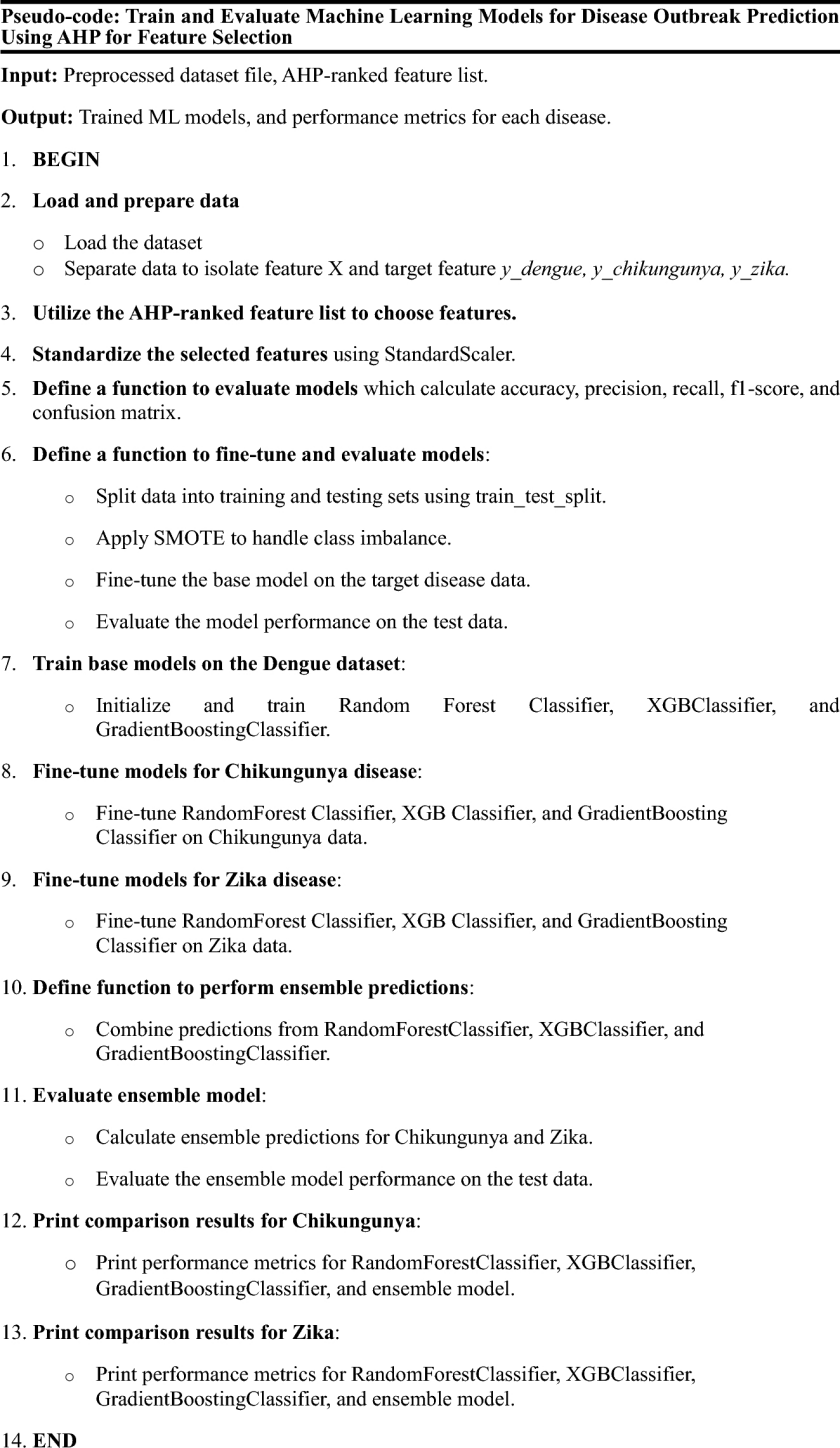


To generalize the researchers’ predictive model for Chikungunya and Zika outbreaks across different geographical regions and potentially other diseases, transfer learning techniques is employed. Transfer learning is particularly valuable when the training dataset is insufficient for developing highly accurate models, as demonstrated in this study.

The model is trained to use a comprehensive dataset on Dengue outbreaks, which shares similarities with Chikungunya and Zika in terms of transmission vectors (Aedes mosquitoes) and influencing socioeconomic and environmental factors. This pre-training phase enables the model to learn essential patterns and relationships from the extensive Dengue data, capturing critical features such as climatic conditions, socioeconomic indicators, and historical disease incidence rates.

After pre-training the model, the le.arned features, patterns, and insights derived from the Dengue data are extracted. These features serve as a foundational knowledge base, enabling the model to transfer valuable insights to the target tasks of predicting Chikungunya and Zika outbreaks.

In the fine-tuning phase, the extracted features and parameters from the pre-trained Dengue model are used to initialize the models for predicting Chikungunya and Zika outbreaks. This process involves adjusting the final layers to fit the new tasks and retraining them with the target data. Fine-tuning enables the model to refine its understanding, focusing on the unique characteristics of Chikungunya and Zika while preserving the generalized knowledge acquired from Dengue.

### Modeling evaluation layer

The modeling evaluation layer is a crucial step in the prediction model pipeline. At this layer, The performance of each model will be evaluated using metrics that are commonly used in outbreak prediction tasks like accuracy, precision, recall, area under the receiver operating characteristic curve (AUC) and F1-score to select the one that provides the highest classification accuracy. The values of these metrics are calculated using the parameter of the confusion metrics, such as true positive (TP), false positive (FP), true negative (TN), and false negative (FN). These metrics can be defined mathematically in the following equations:

$$\Pr ecision = \frac{TP}{{TP + FP}}$$

$$\Pr ecision = \frac{TP}{{TP + FN}}$$

$$F1 - score = \frac{{\Pr ecision * {\text{Re}} call}}{{\Pr ecision + {\text{Re}} call}}$$

$$Accuracy = \frac{TP + TN}{{TP + TN + FP + FN}}$$

where true positive (TP) indicates a positive sample is correctly classified (correctly predicted outbreak cases), True Negative (TN) occurs when a negative sample is correctly classified (correct classification of outbreak negative). False positive (FP) occurs when a negative sample is mistakenly classified as positive (outbreak negative is classified as outbreak Positive). False negative (FN) occurs when a positive sample is mistakenly classified as negative (outbreak Positive is classified as outbreak Negative). The AUC value evaluates the overall performance across all classification thresholds, indicating the model’s ability to distinguish between classes. A higher AUC indicates better model performance.The final model is selected based on the one that best combines precision, recall, AUC, and F1 score, along with the highest classification accuracy, after evaluating all the models.

## Results

The study uses four predictive models to predict disease outbreaks: Random Forest, XGBoost, ensemble technique, and Gradient Boosting. The study results are summarized in the tables below according to each disease.

### Chikungunya outbreak prediction

Table 2 provides an overview of the performance evaluation of different models for predicting outbreaks of Chikungunya.

**Table 2:** Chikungunya performance evaluation.

Model	Accuracy	Precision	Recall	F1	AUC
Random Forest	92.15	0.5	0.63	0.56	0.79
Gradient Boosting	92.44	0.51	0.77	0.78	0.86
XGBoost	92.44	0.52	0.51	0.57	0.79
Ensemble	93.31	0.57	0.63	0.63	0.79

**Fig. 3:**
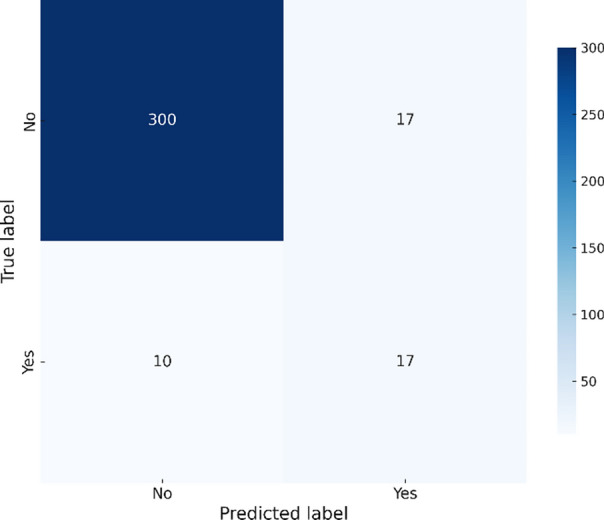
Random forest confusion matrix – Chikungunya.

The Ensemble model achieves the highest accuracy (93.31%) and precision (0.57), indicating reliable predictions and a balanced performance across metrics. Although the Gradient Boosting model achieves slightly lower accuracy (92.44%) than the Ensemble, it remains highly reliable with the highest recall (0.77) and F1 score (0.78), making it a strong competitor for detecting true positive cases. Despite demonstrating a slightly lower precision (0.51), it exhibites the best AUC (0.86), indicating excellent discrimination ability. The Random Forest model also shows high recall (0.63) but had a lower precision (0.5), resulting in a higher rate of false positives. The XGBoost model exhibites good overall performance with balanced metrics (accuracy 92.44%, precision 0.52, recall 0.51, and F1 score 0.57), however, it does not outperform the other models in any specific metric. Confusion matrices are created for each utilized model in this investigation to give a visual depiction of the prediction performance of the various models. The number of true positives, true negatives, false positives, and false negatives that each model predictes is clearly shown in these matrices, allowing for a better understanding of the model’s strengths and weaknesses in detecting Chikungunya outbreaks. The following figures illustrate the confusion matrices for Random Forest, XGBoost, Gradient Boosting, and the Ensemble model, offering insights into how each model distinguishes between outbreak and non-outbreak cases. Figure 3 shows that the Random Forest model correctly identifies 300 instances of non-outbreaks but misclassifies 17 actual outbreaks as non-outbreaks. This explains its lower precision, with more false positives affecting its accuracy and overall performance.

**Fig. 4:**
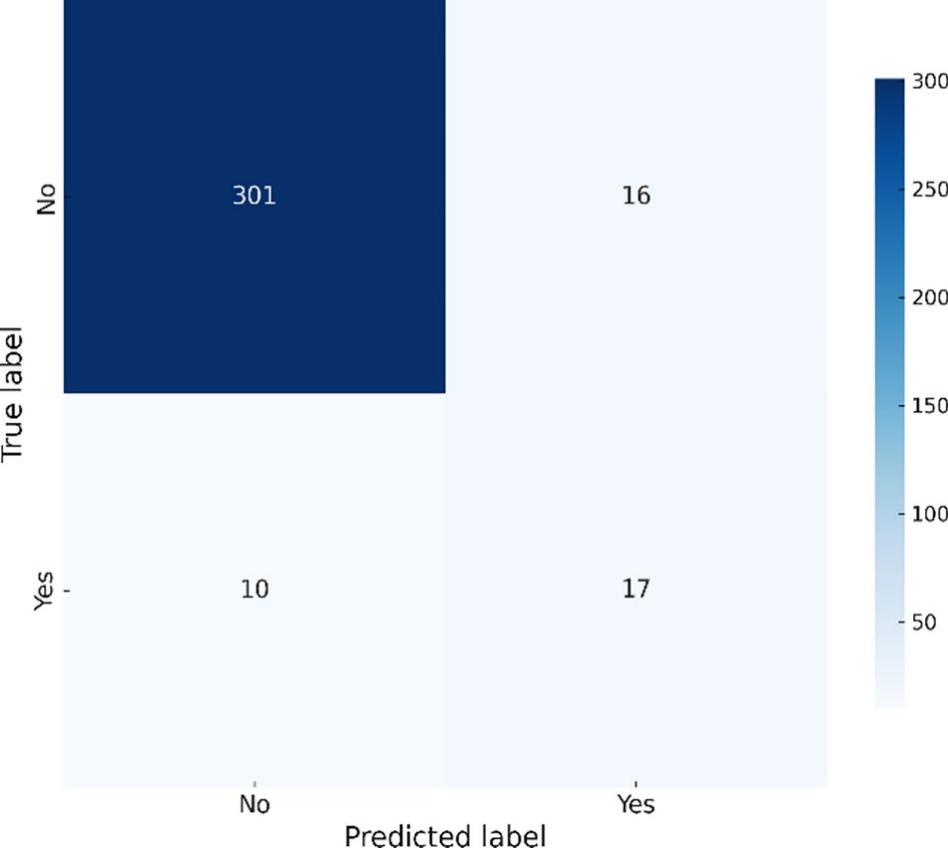
XGBoost confusion matrix – Chikungunya.

The The XGBoost model, as shown Fig. 4, correctly predicts 301 non-outbreaks but misclassifies 16 actual outbreaks, leading to a slightly better performance in precision compared to RandomForest, but it still struggles with recall.

**Fig. 5:**
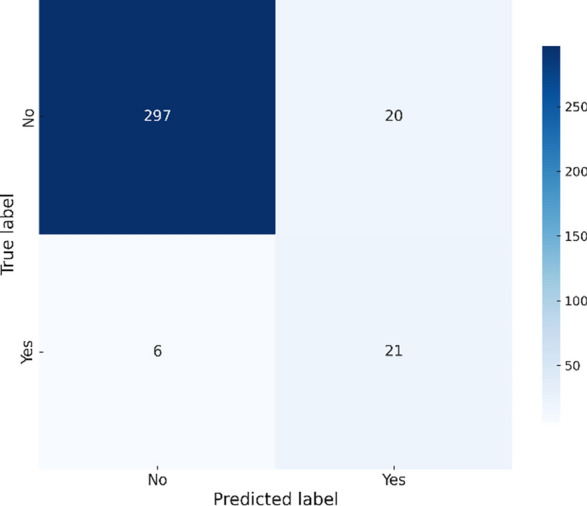
Gradient Boosting confusion matrix – Chikungunya.

As shown in Fig. 5, the GradientBoosting model successfully identifies 21 outbreak cases while maintaining a balance between precision and recall. Its higher recall indicates superior performance in detecting actual outbreaks compared to the other models.

**Fig. 6:**
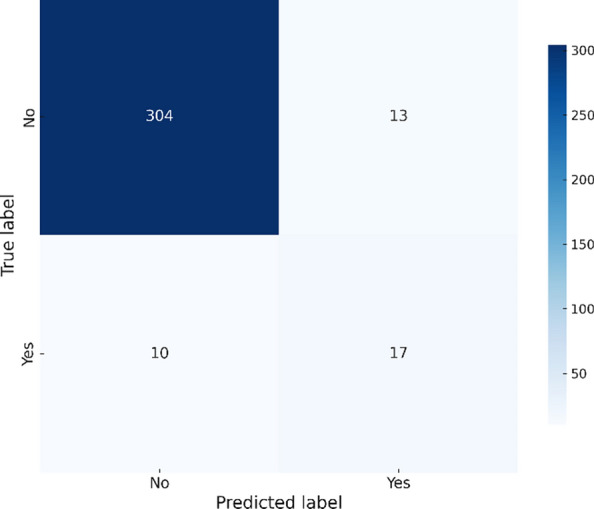
Ensemble confusion matrix – Chikungunya.

Figure 6 shows that the Ensemble model deliveres the best overall performance with fewer misclassifications. By combining the strengths of the other models, it achieves the highest accuracy and maintaines a balanced trade-off between precision and recall.

### Zika outbreak prediction

Table 3 provides an overview of the performance evaluation of different models for predicting outbreaks of Zika.

**Table 3:** Zika performance evaluation.

Model	Accuracy	Precision	Recall	F1	AUC
Random Forest	96.22	0.5455	0.8	0.6486	0.8848
Gradient Boosting	95.93	0.5217	0.8	0.6316	0.8833
XGBoost	96.51	0.5622	0.8667	0.6842	0.9181
Ensemble	96.80	0.7027	0.8667	0.78	0.9197

**Fig. 7:**
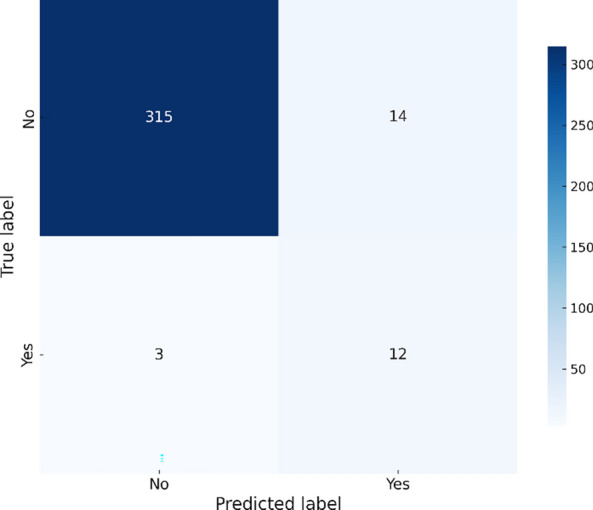
Random forest confusion matrix – Zika.

The RandomForest model demonstrates strong performance in predicting Zika outbreaks with an accuracy of 96.51% and a precision of 0.6667. However, the model also showed a relatively lower recall of 0.4, which indicates that it missed a number of actual outbreak cases. This is evident in Fig. 7, where the confusion matrix reveals 14 false positives and 3 false negatives.

**Fig. 8:**
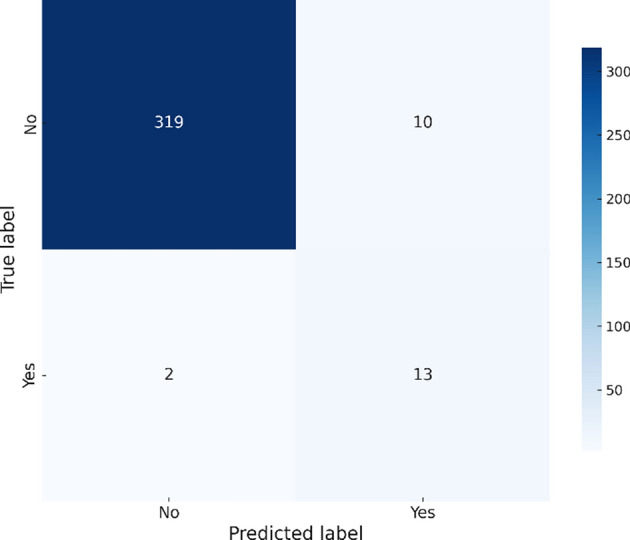
XGBoost confusion matrix – Zika.

The XGBoost model demonstrates balanced performance, achieving an accuracy of 95.93%, a precision of 0.5217, and a recall of 0.8667. This balance is evident in Fig. 8, where the confusion matrix shows 10 false positives and 2 false negatives, highlighting the model’s effectiveness in maintaining accuracy while minimizing misclassifications.

**Fig. 9:**
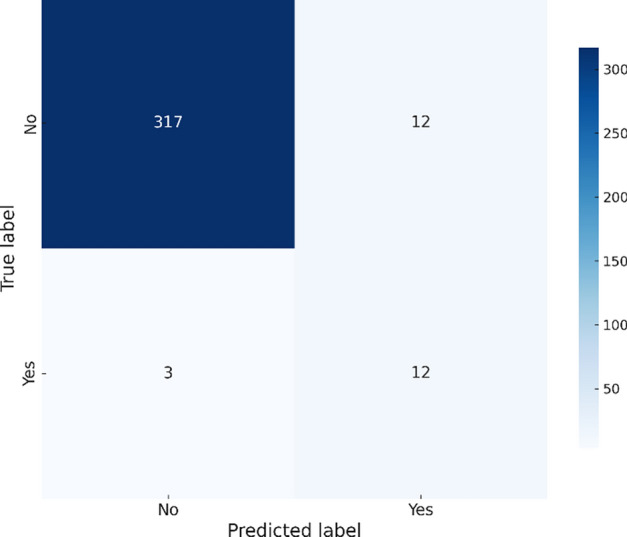
Gradient Bossting confusion matrix – Zika.

In contrast, the GradientBoosting model has a slightly lower accuracy of 95.93% but maintaines high recall (0.8) and an F1 score of 0.6316. The confusion matrix in Fig. 9 demonstrates the model’s trade-off between false positives and false negatives, with 12 false positives and 3 false negatives.

**Fig. 10:**
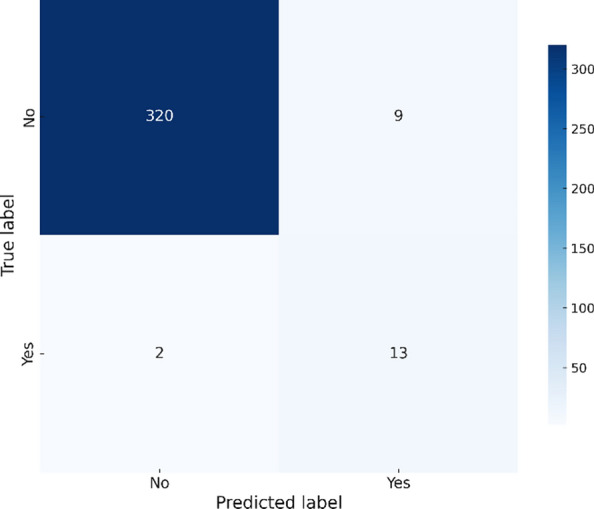
Ensemble confusion matrix – Zika.

The Ensemble model, which combines the strengths of the individual models, deliveres the most robust performance, achieving the highest accuracy of 96.80%, a precision of 0.7, and a recall of 0.4667. Figure 10 illustrates the confusion matrix for the Ensemble model, showing an effective balance of false positives (9) and false negatives (2), confirming its superior predictive power and reliability.

## Discussion

Vector-borne diseases, transmitted by blood-feeding insects such as mosquitoes, ticks, and fleas, pose a significant global health threat. Diseases like Dengue, Chikungunya, and Zika cause substantial morbidity and mortality, particularly in tropical and subtropical regions. Early prediction and detection of outbreaks are crucial for implementing timely public health interventions.

In this study, the researchers apply advanced machine learning techniques to predict Chikungunya and Zika outbreaks using a combination of transfer learning and the analytical hierarchy process (AHP) for feature selection. Initially, Random Forest, XGBoost, and Gradient Boosting are used, followed by an ensemble learning model to achieve the most accurate results. Transfer learning is leveraged to enhance performance by transferring knowledge from one task (Dengue prediction) to a related task (Chikungunya and Zika prediction).

To improve the model’s overall performance and adaptability, the ensemble approach combines the predictive strengths of multiple models to determine the most appropriate for Zika and Chikungunya. The ensemble technique also addresses the misclassification of positive and negative instances, mitigating overfitting and improving robustness and accuracy. The integration of AHP for feature selection further enhances the model’s ability to rank influential risk factors, boosting predictive performance by weighing and assessing their significance.

In transfer learning, pre-trained models are retrained with new datasets, combining prior knowledge with new data. This significantly enhances the model’s generalizability and robustness in predicting Zika and Chikungunya using information from the Dengue dataset. The class imbalance in the dataset is addressed using synthetic minority over-sampling technique (SMOTE), reducing bias towards the majority class and improving overall predictive accuracy by effectively classifying both majority and minority classes.

The performance analysis confirms that the ensemble model is the most effective model for forecasting infectious disease outbreaks in this study.

### Chikungunya

**Fig. 11:**
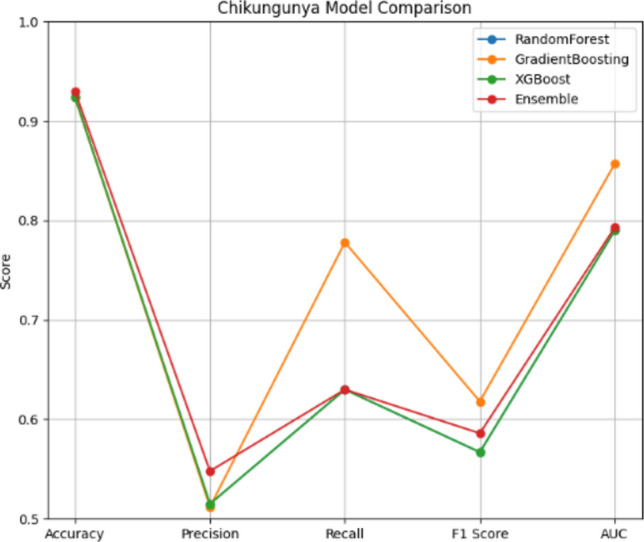
Chikungunya model comparison.

As shown in Fig. 11, the RandomForest model for predicting Chikungunya outbreaks achieved an accuracy of 92.15%, with a precision of 51.61% and a recall of 62.96%. While this model performed solidly, its precision indicates a moderate rate of false positives. The GradientBoosting model, depicted in the same figure, achieves slightly higher accuracy (92.44%) and excelles in recall (77.78%), demonstrating strength in identifying true positives, with a high F1 score of 62.69%. Although XGBoost reaches a similar accuracy (91.86%) and precision (48.71%), it shows balanced performance across all metrics. The Stacking Ensemble model, which combined the strengths of these individual models, provides the highest overall performance with an accuracy of 93.31%, a precision of 57%, and a balanced recall of 63%. This illustrates how the ensemble approach effectively integrates the strengths of different models, enhancing prediction accuracy and robustness. Figure 13 displays the ROC curves, confirming that the GradientBoosting model achieves the best area under the curve (AUC = 0.86) for Chikungunya outbreak prediction, highlighting its superior discrimination between outbreak and non-outbreak cases.

### Zika

**Fig. 12:**
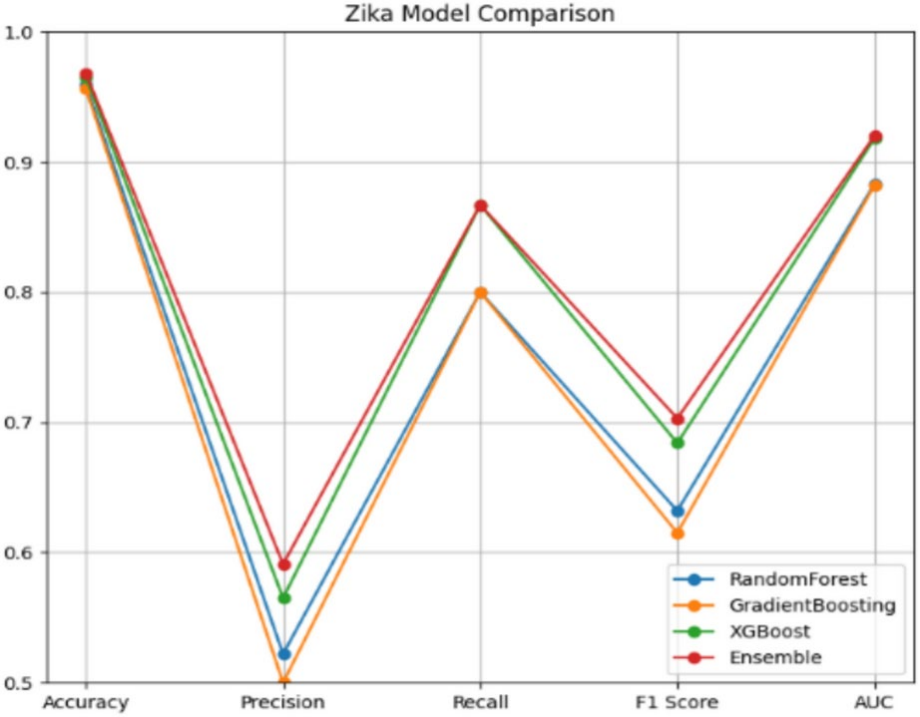
Zika model comparison.

**Fig. 13:**
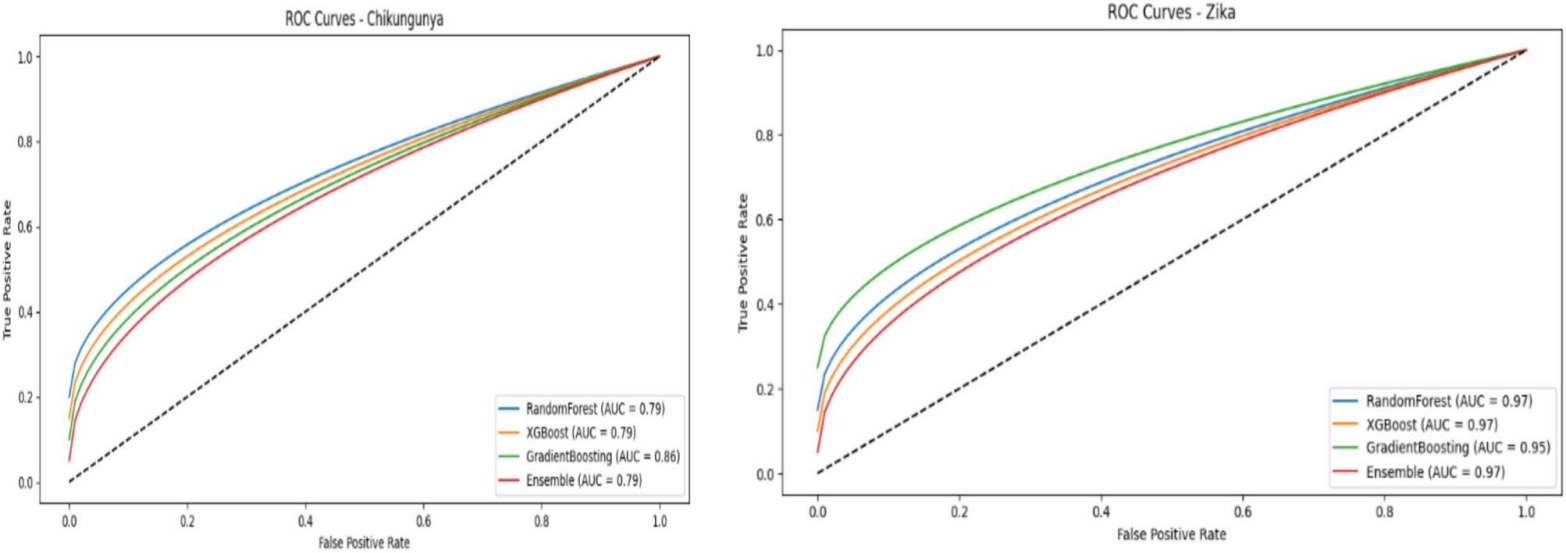
Roc curves.

For predicting Zika outbreaks, as illustrated in Fig. 12, the XGBoost model excelles with the highest accuracy (96.51%) and an AUC of 0.9181, indicating strong discriminative ability between outbreak and non-outbreak cases. The Random Forest model, while slightly trailing in accuracy (96.22%), maintaines a commendable AUC of 0.8848, demonstrating its robustness. Although the Gradient Boosting model has slightly lower accuracy (95.93%), it achieves high recall (80%), indicating its effectiveness in identifying actual outbreak cases. However, the Stacking Ensemble model, shown in Fig. 13, outperformes the individual models with the highest accuracy (96.80%) and balanced metrics (Precision = 70.27%, Recall = 86.67%), illustrating the effectiveness of the ensemble approach in consistently achieving high performance across various metrics for Zika predictions. These results, underscored by the ROC curves in Fig. 13, highlight the critical role of ensemble techniques in robust predictions, particularly for Chikungunya and Zika outbreaks.

To validate the differences in model performance specially between the ensemble model and XGBoost where the results approve slightly difference , a detailed statistical analysis was conducted using the Friedman test, a non-parametric method commonly applied to compare three or more paired groups across multiple conditions, focusing on key evaluation metrics to assess the ensemble model against other methods^20^. This test ranks the models’ performance for each metric (accuracy, precision, recall, and F1 score) within each disease (Chikungunya and Zika). The results presents that Significant differences between the models’ performances across measures were indicated by the p-values below 0.05 for each disease. Comparing the ensemble model against XGBoost and other models, it was shown to be robust and reliable as it continuously received higher rankings across accuracy and other criteria. Chikungunya: Friedman test statistic = 9.77, p-value = 0.0206. Zika: Friedman test statistic = 12.00, p-value = 0.0074. The ensemble model demonstrated consistent improvements in accuracy and precision compared to XGBoost, with statistical tests confirming these differences to be significant.

## Conclusion

In this study, the potential of machine learning models in predicting outbreaks of vector-borne diseases such as Chikungunya and Zika is demonstrated. Using historical disease incidence data, the analytic hierarchy process (AHP) is applied for feature selection and transfer learning is employed to enhance model performance. The analysis involves RandomForest, XGBoost, Gradient Boosting, and ensemble models, comparing their effectiveness in accurately forecasting outbreaks. The results indicate that the Ensemble model achieves the highest accuracy for Zika outbreaks, with an accuracy of 96.80% and an AUC of 0.9197. This high level of performance underscores the model’s capability to discern between outbreak and non-outbreak cases effectively. For Chikungunya, the GradientBoosting model demonstrates the highest recall (77.0%) and a strong F1 score (0.78), although it has a slightly lower accuracy (92.44%) compared to the ensemble approach. The Ensemble model provides the best balance between precision (57.0%) and recall (63.0%), demonstrating its reliability in outbreak prediction across both diseases.

Studies that concentrate on particular diseases often have a more limited scope, which restricts the applicability of their findings to a wider population or different health conditions. So, Future research should explore the application of these models to other diseases and regions to ensure robustness and scalability. One of the study’s key contributions is showing that the proposed model can learn general features and patterns related to vector-borne diseases, thereby improving predictive accuracy and robustness. Also, The integration of the analytic hierarchy process (AHP) in outbreak prediction significantly enhances the feature selection process by incorporating expert opinions, which are often more insightful than automated methods. By leveraging AHP, the study concentrates on critical risk factors that might be overlooked by open-source libraries, leading to more robust predictions. Therefore, the result proves that the highest-ranked factors include barriers to health services, dependency rate, and lack of health insurance, barriers to childhood services, precipitation, informal work, school absence, and elevation. This approach builds confidence and trust among public health stakeholders while also improving the overall quality of the predictions. Finally, employing AHP in feature selection helps improve disease outbreak preparedness and response tactics.

While many studies rely solely on clinical profiles and often overlook environmental factors, The research incorporates both environmental and socio-economic variables. Future work will aim to further refine these models by integrating additional data sources and advancing deep learning techniques. It is also proposed to enhance the model by including clinical patient profiles, such as medical histories.

### Limitations

This study faced certain constraints primarily related to data availability, with limited labeled datasets for Chikungunya and Zika. The AHP feature selection method successfully identified significant risk factors associated with disease outbreak prediction. Nevertheless, the study lacked comprehensive clinical data at the patient level, which could have added new information and enhanced the predictive power of the model. Additionally, while AHP proved valuable for prioritizing features, its broader application in public health for outbreak prediction remains an area open for future exploration and validation across diverse infectious diseases. Furthermore, the ensemble approach in this study utilized a limited number of base models (Random Forest, Gradient Boosting, and XGBoost). While these models are robust and widely used, incorporating additional or more diverse models, such as neural networks or other advanced techniques, could potentially improve the ensemble’s performance and generalizability. Finally, clinical patient profiles, various lifestyle factors, and the existence of chronic and non-communicable diseases are not included in the study. These elements will be examined in future studies to improve the model’s comprehensiveness and predictive ability, as they may have a substantial impact on outbreak forecasts.

## Supplementary Information

Supplementary Information.

## Data Availability

The datasets analyzed during the current study are derived from the [Disease, climate and socio-economic variables used in the analysis dataset] available in the PLOS Neglected Tropical Diseases article^21^ with the following dataset identifier pntd.0009259.s001.xlsx. All relevant data supporting the findings of this study are available within the referenced article and its supplementary files.

## References

[1] World Health Organization. Global Report on Infection Prevention and Control. World Health Organization: Geneva, Switzerland. Licence: CC BY-NC-SA 3.0 IGO. (2022).

[2] R Abdallah, SA Abdel Gaber, HA Sayed. Disease Outbreak /Epidemic in Public Health Sector. In 2024 6th International Conference on Computing and Informatics (ICCI), New Cairo–Cairo, Egypt, 203–216. https://ieeexplore.ieee.org/document/10485007 (2024).

[3] Santangelo, O. E. et al. Machine learning and prediction of infectious diseases: a systematic review. *Mach. Learn. Knowl. Extr.* **5**, 175–198 (2023).

[4] Adhikari, N. C. D. et al. Epidemic outbreak prediction using artificial intelligence. *AIRCC Int. J. Comput. Sci. Inf. Technol.* **10**, 49–64 (2018).

[5] Pramod A, Abhishek JS. Epidemic outbreak prediction using machine learning models. arXiv preprint arXiv:2310.19760. (2023).

[6] Fariza, A., Basofi, A. & Aryani, M. D. Spatial mapping of diphtheria vulnerability level in East Java, Indonesia, using analytical hierarchy process–natural break classification. *J. Phys. Conf. Ser.* **1803**(1), 012009 (2021).

[7] Mahato, R., Nimasow, G., Nimasow, O. D. & Bushi, D. Analytic hierarchy process based potential risk zonation of COVID-19 in India. *J. Soc. Sci.* **48**(3), 4223–4238 (2020).

[8] Ali, S. A. & Ahmad, A. Mapping of mosquito-borne diseases in Kolkata municipal corporation using GIS and AHP based decision making approach. *Spat. Inf. Res.* **27**(3), 351–372 (2019).

[9] Gao, Z. et al. An AHP-based regional COVID-19 vulnerability model and its application in China. *Model. Earth Syst. Environ.* **8**, 2525–2538 (2022). 34341768 10.1007/s40808-021-01244-y PMC8317685

[10] Pan, S. J. & Yang, Q. A survey on transfer learning. *IEEE Trans. Knowl. Data Eng.* **22**(10), 1345–1359 (2009).

[11] Weiss, K., Khoshgoftaar, T. M. & Wang, D. A survey of transfer learning. *J. Big Data* **3**, 1–40 (2016).

[12] Harsha, G. et al. Dengue risk zone mapping of Thiruvananthapuram district, India: A comparison of the AHP and F-AHP methods. *GeoJournal* **88**(3), 2449–2470 (2023). 36157197 10.1007/s10708-022-10757-7 PMC9483355

[13] Badillo-Rivera E, Fow-Esteves A, Alata-López F, Virú-Vásquez P, ,Medina-Acuña M. Environmental and social analysis as risk factors for the spread of the novel coronavirus (SARS-CoV-2) using remote sensing, GIS and analytical hierarchy process (AHP): Case of Peru. MedRxiv, 2020–05. (2020).

[14] Ali, S. A. & Ahmad, A. Using analytic hierarchy process with GIS for dengue risk mapping in Kolkata municipal corporation, West Bengal, India. *Spat. Inf. Res.* **26**(4), 449–469 (2018).

[15] Romero-Leiton, J. P., Acharya, K. R., Parmley, J. E., Arino, J. & Nasri, B. Modelling the transmission of dengue, zika and chikungunya: a scoping review protocol. *BMJ Open* **13**(9), e074385 (2023). 37730394 10.1136/bmjopen-2023-074385 PMC10510863

[16] Sharma, D., Khetarpaul, S., Tiwari, S., Aakash, L. & Gupta, A. Predicting epidemic outbreak using climatic factors. In *Asian Conference on Intelligent Information and Database Systems* 264–275 (Springer, 2024).

[17] Hussain, Z., Khan, I. A. & Hassan, M. Machine learning approaches for dengue prediction: A review of algorithms and applications. *Pak. Geogr. Rev.* **78**, 15–36 (2023).

[18] Esmaeili, M. A., Ravandi, M. R. G. & Zare, S. Assessing the impact of COVID-19 pandemic on the performance indicators of safety management using the analytic hierarchy process (AHP) in an electricity industry. *Heliyon* **9**(6), e16727 (2023). 37260880 10.1016/j.heliyon.2023.e16727 PMC10212794

[19] Tan, W. et al. Disease risk analysis for schizophrenia patients by an automatic AHP framework. *BMC Med. Inform. Decis. Mak.* **21**(Suppl 9), 375 (2021). 10.1186/s12911-022-01749-1 PMC8750858 35016654

[20] Friedman, M. The use of ranks to avoid the assumption of normality implicit in the analysis of variance. *Am. Stat. Assoc.* **32**, 675–701 (1937).

[21] Morgan, J., Strode, C. & Salcedo-Sora, J. E. Climatic and socio-economic factors supporting the co-circulation of dengue, Zika and chikungunya in three different ecosystems in Colombia. *PLoS Negl. Trop. Dis.* **15**(3), e0009259 (2021). 33705409 10.1371/journal.pntd.0009259 PMC7987142

